# Proton-pump inhibitors increase *C. difficile* infection risk by altering pH rather than by affecting the gut microbiome based on a bioreactor model

**DOI:** 10.1080/19490976.2025.2519697

**Published:** 2025-06-16

**Authors:** Julia Schumacher, Patrick Müller, Johannes Sulzer, Franziska Faber, Bastian Molitor, Lisa Maier

**Affiliations:** a Cluster of Excellence EXC 2124 Controlling Microbes to Fight Infections, University of Tübingen, Tübingen, Germany; b Environmental Biotechnology Group, Department of Geosciences, University of Tübingen, Tübingen, Germany; c Interfaculty Institute for Microbiology and Infection Medicine Tübingen, University of Tübingen, Tübingen, Germany; d M3-Research Center for Malignome, Metabolome and Microbiome, University of Tübingen, Tübingen, Germany; e Helmholtz Centre for Infection Research, Helmholtz Institute for RNA‐based Infection Research (HIRI) Würzburg, Würzburg, Germany; f Institute for Hygiene and Microbiology, Julius‐Maximilians‐University of Würzburg, Würzburg, Germany

**Keywords:** Proton-pump inhibitor, gut microbiota, *clostridioides difficile* infection, bioreactor, colonization resistance

## Abstract

*Clostridioides difficile* infections often occur after antibiotic use, but they have also been linked to proton-pump inhibitor (PPI) therapy. The underlying mechanism – whether infection risk is due to a direct effect of PPIs on the gut microbiome or changes in gastrointestinal pH – has remained unclear. To disentangle both possibilities, we studied the impact of the proton-pump inhibitor omeprazole and pH changes on key members of the human gut microbiome and stool-derived microbial communities from different donors *in vitro*. We then developed a custom multiple-bioreactor system to grow a model human microbiome community and a stool-derived community in chemostat mode and tested the effects of omeprazole exposure, pH changes, and their combination on *C. difficile* growth within these communities. Our findings show that changes in pH significantly affect the gut microbial community’s biomass and the abundances of different bacterial taxa, leading to increased *C. difficile* growth within the community. However, omeprazole treatment alone did not result in such effects. These findings imply that the higher risk of *C. difficile* infection following proton-pump inhibitor therapy is probably because of alterations in gastrointestinal pH rather than a direct interaction between the drug and the microbiome. This understanding offers a new perspective on infection risks in proton-pump inhibitor therapy.

## Introduction

*Clostridioides difficile* has become the most common cause of antibiotic-associated diarrhea, ranging from mild to life-threatening colitis.^1^ Antibiotics favor *C. difficile* infections (CDIs) by disrupting the gut microbiome’s protective barrier, creating an environment that promotes spore germination and *C. difficile* growth. While nearly all classes of antibiotics can increase the risk of CDI, the highest risk is associated with broad-spectrum antibiotics, including clindamycin, fluoroquinolones, and cephalosporins.^2–5^ However, *C. difficile* infections can occur without prior antibiotic use.^6–8^ Other factors, such as the use of proton-pump inhibitors (PPIs), have been shown to increase the risk of CDI in several clinical studies.^9–12^ PPIs are indicated for the treatment of conditions like gastroesophageal reflux disease and ulcers.^13^ As such, they are typically used long-term^14^ and are among the most frequently prescribed drugs worldwide, with omeprazole being the most common.^15^ PPIs inhibit the proton/potassium (H^+^/K^+^)-ATPase enzyme in gastric parietal cells, thereby causing an increase in gastric pH. The reasons why PPI consumption is associated with an increased risk of CDI remain unclear.

PPI consumption, similar to antibiotic use, has been linked to alterations in the gut microbiome composition in various studies.^16–18^ These changes include an increase in *Enterococcaceae*, *Lactobacillaceae*, *Micrococcaceae*, *Pasteurellaceae*, *Staphylococcaeceae*, and *Streptococcaceae*, along with a decrease in *Ruminococcaceae*.^10,16,17^ These findings imply that PPIs, like antibiotics, disturb the protective barrier of the gut microbiome, fostering an environment conducive to CDI.^10,17,19,20^ While the direct inhibitory effects of broad-spectrum antibiotics on the gut microbiome have been known for decades,^21^ how PPIs cause changes in the gut microbiome composition remains largely unexplored.

There are two main plausible, not mutually exclusive, explanations for how PPIs affect the microbiome: (1) through direct interaction with gut microbes and (2) by their effect on stomach pH. Recent reports indicate that non-antibiotic drugs can directly inhibit members of the gut microbiome, with an estimated 24% of human-targeted drugs inhibiting the growth of key microbiome members.^22^ By directly targeting gut microbes, PPIs could reduce diversity and shift species abundance, explaining the compositional changes observed in microbiome studies.

Alternatively, the effect of PPIs on the microbiome may be a secondary consequence of changes in gastrointestinal pH. Treatment with PPIs, such as omeprazole, raises the gastric pH above 6 and increases the pH of the proximal duodenum.^23,24^ Specifically, one hypothesis for how PPIs promote CDI is that raising the pH in the stomach lifts the pH barrier of the stomach. This allows *C. difficile* to more easily translocate to the lower intestinal tract. In addition, other bacteria may pass this barrier, which may contribute to shaping the gut microbiome in a *C. difficile*-favorable manner.^13^ However, the pH-increasing effect diminishes in the distal duodenum, and the pH normalizes when reaching the proximal jejunum.^23,25^ Although the pH change in the stomach is not thought to affect later parts of the intestinal tract, PPI treatment could still lead to a pH change in the colon. Colonocytes express a homolog of the H^+^/K^+^-ATPase found in gastric parietal cells, and omeprazole has been proposed to inhibit this enzyme, as well. This could increase pH levels within the colon and stool of individuals using PPIs.^23,26–29^ Notably, CDI has been linked to more alkaline stool.^30^ It is tempting to speculate that the more alkaline colon environment created by PPI treatment may promote *C. difficile* growth, thereby increasing the risk of infection.

In this study, we sought to understand how omeprazole influences the microbiome composition to promote *C. difficile* growth. We focused on determining whether these effects are solely mediated by the drug itself or whether changes in pH play a role. In humans and animal models these effects are interconnected and, therefore, difficult to separate. Thus, we employed various *in vitro* systems, ranging from batch cultivation to bioreactor systems, to precisely quantify the consequences of pH changes and physiological omeprazole concentrations on defined and human-stool-derived gut microbial communities. Subsequently, we challenged pH- and drug-perturbed communities with *C. difficile* and monitored its growth within these communities.^31^ Our findings provide evidence that the increase in *C. difficile* growth associated with omeprazole is primarily a result of pH changes rather than direct interference of the drug with gut microbes.

## Results

### In monoculture, key members of the human gut microbiome respond to pH change but not to omeprazole

To distinguish between the direct impact of PPIs on the human gut microbiome and the effect of altered gastrointestinal pH, we investigated the PPI omeprazole and the pH sensitivity of key microbiome members. Recognizing that gut microbes are most sensitive to perturbation when grown in monoculture,^32^ which is due to the lack of cross-protection found in communities, we first examined the effects of pH and the drug in monocultures. We selected 21 prevalent and abundant members of the human gut microbiome, which can be studied both in monoculture and as part of a community, referred to as Com21 (Suppl. Table S1). These 21 bacterial species represent 5 bacterial phyla, 11 families, and 18 genera, covering 68.6% of the metabolic pathways detected in the human microbiome.^31^

**Figure 1.**
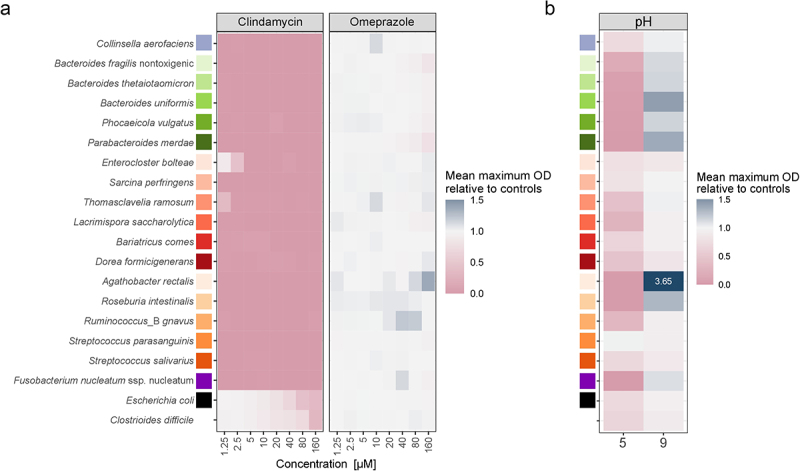
Individual sensitivity of 19 Com21 members to omeprazole and pH. a) Growth of Com21 members in the presence of different concentrations of clindamycin and omeprazole in monoculture. Heatmap depicts the mean maximum optical density (OD) of cultures in the stationary phase compared to untreated controls (*N* = 3). b) Growth of Com21 members at different pH in monoculture. Heatmap depicts the mean maximum OD of cultures in the stationary phase compared to OD at pH 7.4. Values outside the legend range are written within the heatmap tile (*N* = 3).

To assess omeprazole sensitivity, we reanalyzed growth curves^31^ of these strains in modified Gifu Anaerobic Broth (mGAM) in the presence of varying omeprazole concentrations. We also examined the sensitivity of these strains to clindamycin, an antibiotic that is associated with a high risk for CDI.^2–5^ We quantified drug sensitivity by calculating the relative growth in the presence of the drug compared to an untreated control based on the maximum optical density (OD) in the stationary phase. As expected, clindamycin completely inhibited 15 of the 19 tested strains at the lowest concentration of 1.25 µM (Figure 1(a)). *C. difficile* showed the highest resistance to clindamycin, maintaining growth with a relative mean OD of 0.71 at 80 µM and 0.31 at 160 µM compared to untreated controls. *Escherichia coli* also tolerated higher clindamycin concentrations, with relative mean ODs of 0.67 at 40 µM and 0.43 at 80 µM. Additionally, *Enterocloster bolteae* (relative OD of 0.87 at 1.25 µM and 0.44 at 2.5 µM) and *Thomasclavelia ramosa* (relative OD of 0.35 at 1.25 µM) were able to grow at the lowest clindamycin concentrations. In contrast, omeprazole did not significantly affect the growth of any of the Com21 members or *C. difficile*; only at 160 µM did some *Bacteroidales* show a slight reduction in OD (relative mean OD 0.78–0.9) (Figure 1(a)). These results indicate that the PPI omeprazole does not directly inhibit commensal bacterial growth. Therefore, unlike clindamycin, the increased risk for CDI associated with omeprazole is likely not due to growth inhibition of gut bacteria.

Next, we investigated the pH sensitivity of the strains. The normal pH in the gastrointestinal tract varies depending on the intestinal site, diet, gender, and health status but generally falls between 5.5 and 7.5.^23,33–36^ However, it can reach pH 8 to 9 in extreme cases.^35^ We quantified pH sensitivity at pH 5 and pH 9 by measuring the maximum OD in the stationary phase and normalizing it to growth at pH 7.4. Overall, pH 5 had a more severe impact on growth, with several species being unable to grow at this pH (e.g., all *Bacteroidales*; Figure 1(b)). Only *Streptococcus parasanguinis* was unaffected at pH 5. Growth at pH 9 was less impaired, with all strains being able to grow. Some species, such as the *Bacteroidales*, *Agathobacter rectalis*, and *Roseburia intestinalis*, even grew better at pH 9 compared to pH 7.4 (Figure 1(b)).

Our data shows that pH sensitivity varies across species, with lower pH (pH 5) more severely inhibiting bacterial growth. This underscores the importance of low pH in the upper small intestine and stomach as a barrier to incoming bacteria – a barrier that is compromised during long-term PPI treatment, potentially leading to small intestinal bacterial overgrowth.^13^ At pH 9, we observed some growth improvement but generally more growth impairment, indicating that pH changes can affect community structure, abundance, and functions. Thus, our results in monocultures suggest that pH has a more severe impact on individual community members than omeprazole.

### Batch-cultivated human stool-derived communities are insensitive to omeprazole and subsequent C. difficile challenge

Monocultures of selected gut bacterial species fail to fully capture the species diversity, interspecies variation, and individual compositional differences that characterize gut microbiomes. Therefore, we examined microbial communities derived from human fecal samples of healthy donors to assess their sensitivity to omeprazole. Subsequently, we exposed omeprazole-treated communities to *C. difficile* and quantified its growth in these communities.

**Figure 2.**
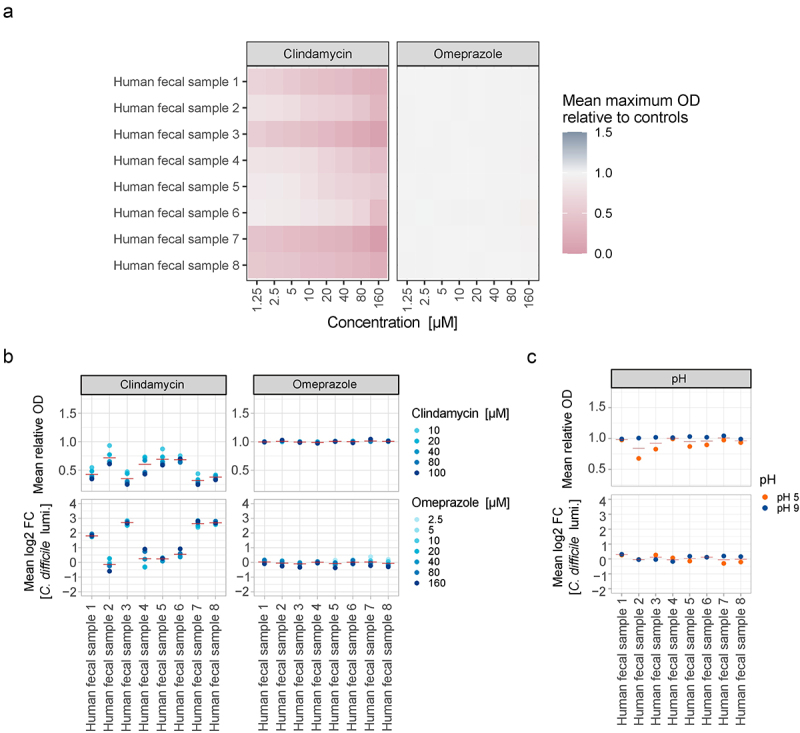
Neither omeprazole treatment nor changes in pH promote the growth of *C. difficile* within human stool-derived microbial communities. a) Growth of communities derived from eight human fecal samples in the presence of different concentrations of clindamycin (left) and omeprazole (right). Heatmap depicts the mean maximum optical density (OD) of cultures in the stationary phase compared to untreated control growth (*N* = 3). b) Top: mean OD of the eight communities relative to untreated controls after treatment with different concentrations of clindamycin (left) or omeprazole (right) for 24 h. Red horizontal line depicts the mean per stool-derived community across all concentrations (*N* = 3) bottom: mean log2 Fold change (FC) of *C. difficile* growth as determined by *C. difficile* luminescence after 5 h in clindamycin (left) or omeprazole (right) treated communities relative to untreated controls. Red horizontal line depicts the mean per stool-derived community across all concentrations (*N* = 3). c) Top: mean OD of eight human stool-derived communities relative to controls at pH 7.4 after growth at different pH for 24 h. Red horizontal line depicts the mean per community (*N* = 3) bottom: mean FC of *C. difficile* growth as determined by *C. difficile* luminescence after 5 h in pH-exposed communities relative to controls at pH 7.4. Red horizontal line depicts the mean per stool-derived community (*N* = 3).

First, we reanalyzed growth curves for omeprazole sensitivity of these human stool-derived communities.^31^ Consistent with single-species sensitivities, the growth of all stool-derived communities was unaffected by omeprazole at all tested concentrations (Figure 2(a)). In contrast, clindamycin sensitivity varied between donors. For example, the community from human fecal sample 5 still grew to a relative mean OD of 0.54 at the highest clindamycin concentration of 160 µM. In comparison, the community from human fecal sample 7 was already reduced to a relative mean OD of 0.46 at the lowest concentration of 1.25 µM (Figure 2(a)). This highlights the inherent differences in antibiotic sensitivities among human gut microbiomes.

Furthermore, we investigated whether omeprazole exposure affects *C. difficile* growth within stool-derived communities. We exposed the communities to various concentrations of omeprazole (2.5 µM to 160 µM) for 24 hours before challenging them with *C. difficile* carrying a constitutive plasmid-based luminescence reporter (Suppl. Figure S1A). The untreated stool-derived communities were able to significantly reduce *C. difficile* growth after 5 hours compared to *C. difficile* grown in monoculture, as measured by luminescence (mean relative *C. difficile* growth in communities: 1.79% ± 0.1 standard error of the mean (SEM)) (Suppl. Figure S1B). Consistent with the omeprazole sensitivity data, the biomass/OD of the community after 24 hours of omeprazole exposure did not change relative to unperturbed controls (Figure 2(b)). Additionally, omeprazole did not impact *C. difficile* growth (Figure 2(b), bottom right).

As a positive control, we conducted the same challenge with clindamycin-treated stool-derived communities (10 µM to 100 µM). Clindamycin reduced community biomass to varying degrees among donors in a concentration-dependent manner (Figure 2(b)). Consistent with clinical observations, clindamycin also increased *C. difficile* growth after pathogen challenge in at least four of the eight samples, up to 9-fold (Figure 2(b), bottom left). Overall, communities that were more sensitive to clindamycin exhibited higher levels of *C. difficile*, indicating that reduced biomass correlates with increased *C. difficile* growth (Pearson’s ρ = −0.8612, p-value = 9.986e-13).

We also investigated the effect of pH changes on stool-derived communities. The communities were grown at pH 5, pH 9, or physiological pH 7.4 for 24 hours before being challenged with *C. difficile*. Growth at pH 5 reduced the biomass of some fecal communities to a minimum relative OD of 0.68 (Figure 2(c), top). However, neither pH 5 nor pH 9 affected subsequent *C. difficile* growth within the communities (Figure 2(c), bottom).

Since PPIs are typically used long-term, we next investigated prolonged exposure to the drug and altered pH in a continuous dilution experiment in which we subcultured the stool-derived samples every 24 h by diluting 1:100 into fresh medium at either pH 7.4, altered pH, or pH 7.4 with 80 µM omeprazole. For this experiment, we chose more physiologically altered pH values (pH 6 and pH 8) rather than the extremes of pH 5 and pH 9 that we tested before. We measured community biomass and *C. difficile* community growth daily for three days, followed by a 24 h recovery phase at pH 7.4 without treatment (Suppl. Figure S2). Neither continuous exposure to omeprazole nor pH 6 or pH 8 showed any impact on community biomass as measured by OD (Suppl. Figure S2, top) or any major influence on *C. difficile* luminescence upon pathogen challenge (Suppl. Figure S2, bottom).

These results indicate that neither the PPI omeprazole nor pH changes directly affected *C. difficile* growth in stool-derived communities from different donors *in vitro*. However, it is important to note that pH adjustments were made only at the beginning of the experiment, and that the pH is most likely not stable over the full period of time in these uncontrolled batch cultivations. Because we cannot control for a stable altered pH of pH 6 or pH 8 in these assays, we implemented a bioreactor system that offers a scalable and more controllable option to test for individual environmental parameters.

### Multiple-bioreactor system enables precise studies of microbiome perturbations

**Figure 3.**
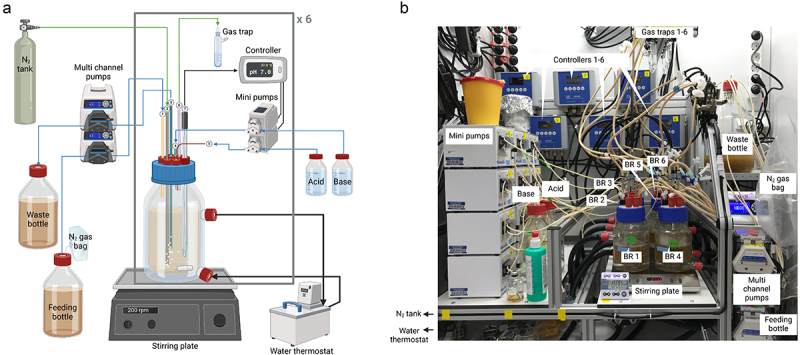
Overview of the multiple-bioreactor system. a) Schematic overview of a single bioreactor bottle. Each double-walled bioreactor bottle has a volume of 500 mL. 1: fresh medium is introduced at a desired flow rate through the feeding port from a feeding bottle; 2: the desired gas mix is introduced into the bottle through a stainless-steel tube with an attached sparger; 3: spent medium is removed through a stainless-steel tube and collected in a waste bottle. This port is also used as a sampling port where samples can be taken with a syringe; 4: a syringe punched through a rubber stopper is used as base port; 5: the acid port is a button once cannula punched through the same rubber stopper as the base port; 6: the gas-out port is connected to a foam trap; 7: an autoclavable pH/pt1000-electrode measuring the pH and temperature is connected to a multi-parameter controller. Each controller is connected to two mini-pumps that are triggered to pump acid or base when the pH falls out of range. All bioreactor bottles are placed on a multi-stirrer plate and stirred with a magnetic stirrer. Temperature is maintained by a water jacket connected to a water thermostat. As indicated by the brackets and the x6 we have 6 bioreactors which can be used simultaneously. Created with BioRender.com. b) Picture of the complete setup. The water thermostat and the gas line are not visible. BR: bioreactor.

To overcome the limitations of working with stool-derived communities in batch, we turned to chemostats and our gut model community Com21. Chemostats allow precise, continuous adjustment and monitoring of environmental conditions, such as pH, over long periods, making them the ideal system to address our question. We chose a previously described system^37^ based on six bioreactor bottles (Figure 3(a,b)), which can be operated simultaneously, individually at different conditions, or as replicates. Our multiple-bioreactor system (MBS) can be operated under an aerobic or anaerobic atmosphere and can be used for batch cultivation or in chemostat mode, where fresh medium is continuously supplied and spent medium is removed at the same rate.

To demonstrate the functionality and reproducibility of our MBS with a gut microbial community, we conducted a pilot experiment in three bioreactors using a reduced version of Com21,^31^ here referred to as Com18. Com18 lacks *E. coli*, *Veillonella parvula*, and *Eggerthella lenta* due to concerns about *E. coli* dominance and initial difficulties in growing *Veillonella* and *Eggerthella* species. The MBS was operated anaerobically for 188.75 hours, starting with 24 hours of batch mode followed by continuous operation in chemostat mode. The OD increased during continuous operation until the 50-hour mark, after which it stabilized (Suppl. Figure S3A). We noted fluctuations in the bioreactor volumes among the individual replicates, which might explain the variations in OD. The pH remained stable at 7 (±0.259) throughout the experiment. The community composition was determined by 16S rRNA gene sequencing (Suppl. Figure S3B).

After 3.25 hours of batch mode, the community was dominated by *Sarcina perfringens* and *Streptococcus salivarius* (Suppl. Figure S3B). By the end of the batch phase at 22 hours, *Fusobacterium nucleatum*, *Bacteroides thetaiotaomicron*, and *Phocaeicola vulgatus* became more dominant, while the relative abundance of *S. perfringens* declined and *S. salivarius* was not detectable anymore. After 22 hours, we proceeded in continuous operation. Here *Bacteroides uniformis* and *F. nucleatum* further increased in relative abundance, while *S. perfringens*, *P. vulgatus*, *R. intestinalis*, and *S. parasanguinis* decreased, despite the overall biomass (OD) remaining constant (Suppl. Figure S3A and B). *R. intestinalis* was lost in all three bioreactors at 116.75 hours. At 188.75 hours, *A. rectalis* was only present in bioreactor 1 and *Ruminococcus gnavus* only in bioreactor 2 (Suppl. Figure S3B). No contamination with non-Com18 species was observed with sequencing and the community composition and biomass of all three replicates were similar (Suppl. Figure S3A and S3B). This pilot experiment demonstrated the suitability of our MBS and evaluation methods for studying microbial communities under controlled conditions.

### In the MBS, pH changes promote C. difficile growth in Com21, whereas omeprazole treatment does not

**Figure 4.**
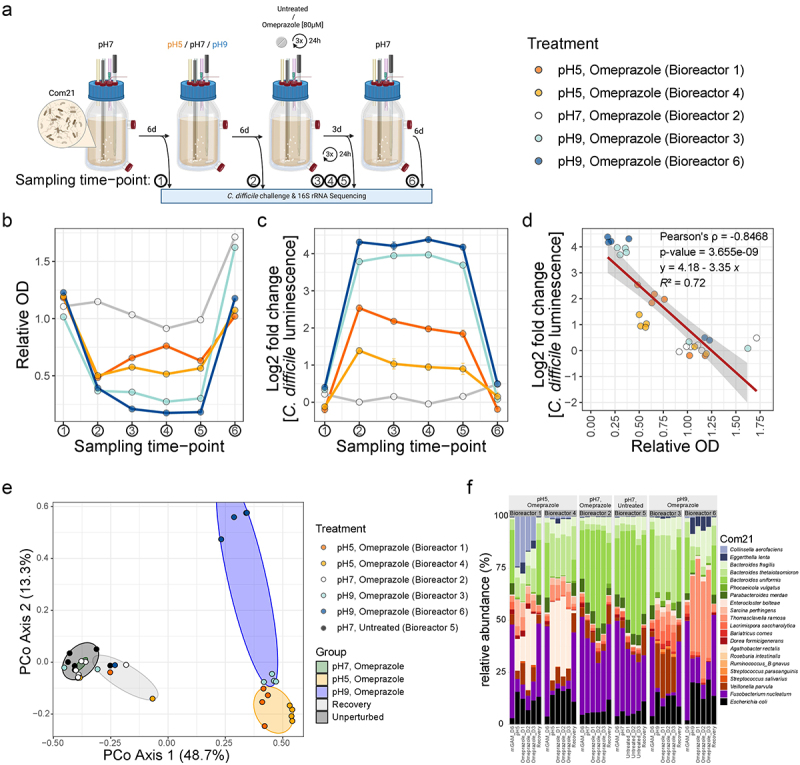
Changes in pH decrease community biomass, significantly alter community composition, and increase growth of *C. difficile* in Com21. a) Schematic overview of the bioreactor workflow. Com21 was grown for six HRTs in chemostat mode with mGAM at pH 7. After this period, the pH was either changed to pH 5 or pH 9 for six HRTs or left unchanged. Subsequently, omeprazole was added daily at 80 µM to five of the six bioreactors for three consecutive HRTs, after which all bioreactors were returned to pH 7 for another six HRTs. Sampling points are indicated with arrows. Created with BioRender.com. b) Relative OD of the bioreactors over time at every sampling time point compared to the median OD of the untreated control (bioreactor 5). Bioreactors 1 and 4 were switched to pH 5, bioreactors 3 and 6 to pH 9, and bioreactor 2 remained at pH 7. All bioreactors, except the control bioreactor 5 used for normalization, underwent omeprazole treatment at 80 µM. c) Log2 Fold change in *C. difficile* growth in the bioreactor communities at every time point. *C. difficile* growth was quantified by luminescence measurement after 5 h and normalized to the median luminescence of *C. difficile* in the untreated control (bioreactor 5) at the same time point. The mean with standard deviation of 10 technical replicates is shown. d) Correlation of relative community OD to log2 Fold change in *C. difficile* growth. Values from plots B and C are shown with colors indicating the corresponding bioreactor. The red line represents the linear trendline, with its function, *R*^2^ value, Pearson correlation, and p-value provided in the plot. e) Principal coordinate analysis of bray-curtis dissimilarity. Data points are color-coded by bioreactor and grouped by treatment (colored ellipses). The untreated control (bioreactor 5), initial compositions of all bioreactors, and pH 7 treatment of bioreactor 2 are grouped as ‘unperturbed’. The three omeprazole treatment sampling points of bioreactor 2 are grouped as ‘pH7, omeprazole’. Sampling points at pH 5 (with and without omeprazole) for bioreactors 1 and 4 are grouped as ‘pH 5, omeprazole’. Sampling points at pH 9 (with and without omeprazole) for bioreactors 3 and 6 are grouped as ‘pH 9, omeprazole’. All recovery sampling points (except bioreactor 5) are grouped under ‘recovery’. f) Relative abundance of each member of Com21 at the indicated sampling time points. Panels are grouped by bioreactor: bioreactors 1 and 4 were changed to pH 5 with omeprazole, bioreactors 3 and 6 were changed to pH 9 with omeprazole, bioreactor 2 was treated with omeprazole, and bioreactor 5 was left untreated.

Next, we used the MBS with Com21 to investigate the effects of pH changes or exposure to omeprazole on the subsequent growth of *C. difficile* (Figure 4). We allowed Com21 to stabilize for six days, which corresponds to six hydraulic retention times (HRTs), before adjusting the pH to 5 (bioreactors 1 and 4), 9 (bioreactors 3 and 6), or 7 (bioreactors 2 and 5). Following an additional six days at the respective pH levels, five out of the six bioreactors (excluding control bioreactor 5) were treated with 80 µM omeprazole for three consecutive days (every 24 hours), based on its estimated concentration in the human intestine.^22^ Afterwards, all bioreactors were set to recover at pH 7 for six days. Samples for the pathogen challenge assay and 16S rRNA gene sequencing were collected after stabilization, pH changes, each day of omeprazole treatment, and after recovery, totaling six samples per bioreactor (Figure 4(a)). At each sampling point, samples were challenged with *C. difficile* using the same assays described for the stool-derived communities.

The untreated Com21 community from bioreactor 5 strongly inhibited *C. difficile* growth to levels observed for stool-derived communities (mean relative *C. difficile* growth in Com21: 1.58% ± 0.19 SEM) (Suppl. Figure S1B). Initially, after six days at pH 7, all bioreactor communities exhibited comparable biomass and similar protection against *C. difficile* challenge, which remained consistent throughout the 21-day experiment in the control bioreactor (Figure 4). When bioreactor 2‘s community, maintained at pH 7, was exposed to 80 µM omeprazole, there was no observable change in OD or *C. difficile* growth, indicating that omeprazole did not directly impact Com21 in our setup (Figure 4(b,c)). This is in line with our observation that omeprazole did not strongly affect the growth of Com21 members in monocultures (Figure 1(a)).

However, altering the pH of the bioreactors to either pH 5 (bioreactors 1 and 4) or pH 9 (bioreactors 3 and 6) resulted in reduced biomass and increased *C. difficile* growth in those communities compared to the control (up to a 22-fold increase in *C. difficile*, Figure 4(b,c)). Notably, omeprazole treatment of communities in altered pH did not further impact their biomass or *C. difficile* growth. Overall, we observed a negative correlation (Pearson’s ρ = −0.8468, p-value = 3.655e-09) between the biomass of the community and the growth of *C. difficile* in this community (Figure 4(d)), similar to what we observed for clindamycin in stool-derived communities (Figure 2(b)).

The changes in pH were accompanied by strong shifts in microbial community composition, while omeprazole treatment alone did not induce any changes (Figure 4(e)). Both pH 5 and pH 9 resulted in decreased levels of *F. nucleatum* and *B. uniformis*, the two most dominant species in the bioreactor communities (Figure 4(f)). Communities at pH 5 showed increased levels of *A. rectalis* and either *Collinsella aerofaciens* (bioreactor 1) or *B. thetaiotaomicron* (bioreactor 4), whereas communities at pH 9 exhibited increased levels of either *B. thetaiotaomicron* and *V. parvula* (bioreactor 3) or *E. lenta* and *T. ramosa* (bioreactor 6). *A. rectalis* was initially absent from all bioreactors but was detected in pH 5-treated bioreactors, where it persisted in low amounts after recovery. Similarly, *S. perfringens* was initially present in low amounts in only one bioreactor but appeared in both pH 5 bioreactors and one pH 9 bioreactor (Figure 4(f)).

These changes in composition were only partially explained by the individual pH sensitivities of the strains (Figure 1(b)). For instance, *Bacteroidales* were found to be acid sensitive, and indeed, their relative abundance decreased in the bioreactors at pH 5. Conversely, *S. perfringens* showed relatively greater resistance to acidity compared to other members, resulting in its increased relative abundance in the pH 5 bioreactors. However, strains sensitive to acidity, such as *A. rectalis*, increased in the pH 5 bioreactors but not in the pH 9 bioreactors, despite demonstrating substantially better growth at pH 9 in monoculture. Additionally, *S. parasanguinis*, which exhibited acid resistance, was absent from all bioreactors initially and did not increase at pH 5.

**Figure 5.**
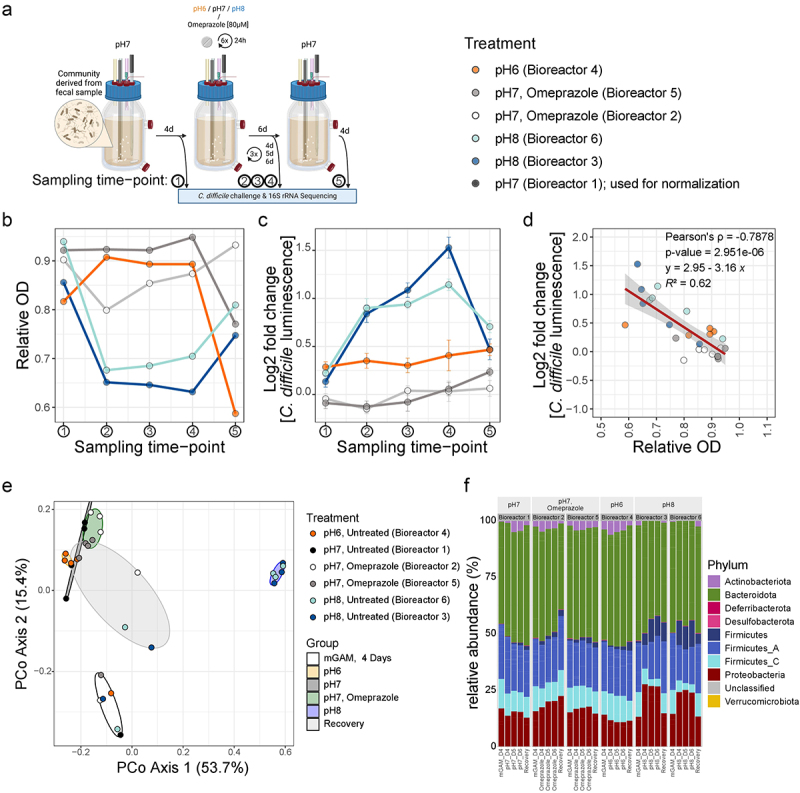
Changes in pH decrease community biomass, alter community composition, and increase growth of *C. difficile* in human stool-derived communities. a) Schematic overview of the bioreactor workflow. A human stool-derived community was grown for four HRTs in chemostat mode with mGAM at pH 7. After this period, the pH was either changed to pH 6 or pH 8 for six HRTs or left unchanged with and without addition of 80 µM omeprazole every 24 h. After the six HRTs, all bioreactors were returned to pH 7 for four HRTs. Sampling points are indicated with arrows. Created with BioRender.com. b) Relative OD of the bioreactors over time at every sampling time point compared to the median OD of the untreated control (bioreactor 1). Bioreactor 4 was switched to pH 6, bioreactors 3 and 6 to pH 8, and bioreactors 2 and 5 remained at pH 7 with the addition of 80 µM omeprazole daily. c) Log2 Fold change in *C. difficile* growth in the bioreactor communities at every time point. *C. difficile* growth was quantified by luminescence measurement after 5 h and normalized to the median luminescence of *C. difficile* in the untreated control (bioreactor 1) at the same time point. The mean with standard deviation of 10 technical replicates is shown. d) Correlation of relative community OD to log2 Fold change in *C. difficile* growth. Values from plots B and C are shown with colors indicating the corresponding bioreactor. The red line represents the linear trendline, with its function, R^2^ value, Pearson correlation, and p-value provided in the plot. e) Principal coordinate analysis of bray-curtis dissimilarity. Data points are color-coded by bioreactor and grouped by treatment (colored ellipses). Initial compositions of all bioreactors are grouped as ‘mGAM, 4 days’. The untreated bioreactor 1 is grouped as ‘pH 7’. The three omeprazole treatment sampling points of bioreactor 2 and 5 are grouped as ‘pH7, omeprazole’. Sampling points at pH 6 for bioreactor 4 are grouped as ‘pH 6’. Sampling points at pH 8 for bioreactors 3 and 6 are grouped as ‘pH 8’. All recovery sampling points (except bioreactor 1) are grouped under ‘recovery’. f) Relative abundance (at phylum level) of human stool-derived sample 1 in the bioreactors at the indicated sampling time points. Panels are grouped by bioreactor: bioreactor 1 was left untreated, bioreactors 2 and 5 were treated with omeprazole, bioreactor 4 was changed to pH 6, and bioreactors 3 and 6 were changed to pH 8.

Remarkably, all bioreactor communities reverted to their original biomass, protection against *C. difficile* growth, and community composition after recovery at pH 7 for six HRTs (Figures 4 and 5). In summary, these findings indicate that a shift in pH modifies the composition of the human gut microbial community, resulting in decreased biomass and reduced resistance to *C. difficile* growth. Importantly, omeprazole does not induce such changes on its own, suggesting that the reported association between PPI usage and an increased risk of CDI could be attributed to the prolonged alterations in the pH of the gastrointestinal tract caused by the drug rather than its direct interaction with gut microbes.

### Growth of C. difficile in a human stool-derived community in the MBS is increased at pH 8 but not at pH 6 or with omeprazole treatment

Com21 is a model community that cannot fully recapitulate the complexity of the human gut microbiome. In contrast, the eight different human stool-derived communities represent more complex communities comprising many different phyla (Suppl. Figure S4A). Their overall phylum compositions were comparable, and alpha-diversities (Shannon Index) were moderately high ranging from 2.2 (Human fecal sample 8) to 3.2 (Human fecal sample 1). For this reason, we decided to investigate the human stool-derived community from human fecal sample 1 with our MBS. Similar to the previous continuous dilution experiment, we chose more physiological pH alterations for this setup, i.e., pH 6 and pH 8 (Figure 5(a)). Furthermore, we separated the pH alterations from omeprazole treatment.

We allowed the stool-derived community to stabilize for four days, which corresponds to four HRTs, before adjusting the pH to 6 (bioreactor 4), pH 8 (bioreactors 3 and 6), or treating with 80 µM omeprazole at pH 7 (bioreactors 2 and 5). Bioreactor 1 was left at pH 7 and untreated and functioned as the control. All bioreactors were kept at their respective pH for six days, with bioreactors 2 and 5 being supplemented with 80 µM omeprazole every 24 h. Afterwards, all bioreactors were set to recover at pH 7 for four days. Samples for the *C. difficile* challenge assay and 16S rRNA gene sequencing were collected after stabilization, four, five, and six days of pH change or omeprazole treatment, and after recovery, totaling five samples per bioreactor (Figure 5(a)). At each sampling point, samples were challenged with *C. difficile* using the same assays described for the Com21 bioreactor samples.

The untreated human stool-derived community from bioreactor 1 strongly inhibited *C. difficile* growth (mean relative *C. difficile* growth in stool-derived community: 1.38% ± 0.04 SEM) (Suppl. Figure S1B). After four days at pH 7, all bioreactor communities exhibited comparable biomass and similar protection against *C. difficile* challenge, which remained consistent throughout the 14-day experiment in the control bioreactor 1. The two bioreactors treated with 80 µM omeprazole (bioreactors 2 and 5) showed only a small decrease in biomass and no major change in *C. difficile* growth, indicating that omeprazole did not directly impact the stool-derived communities in ways that impact these readouts (Figure 5(b,c)). This is in line with our previous observation that omeprazole did not strongly affect the stool-derived communities *in vitro* (Figure 2 and Suppl. Figure S2) or Com21 in the bioreactor (Figure 4). Additionally, altering the pH to 6 (bioreactor 4) did not affect biomass during treatment. Only after recovery did the OD decrease compared to the control (Figure 5(b)). Still, this was not accompanied by a substantial increase in *C. difficile* growth (Figure 5(c)).

However, altering the pH of the bioreactors to pH 8 (bioreactors 3 and 6) resulted in reduced biomass and increased *C. difficile* growth in those communities compared to the control (up to a 3.3-fold increase in *C. difficile*, Figure 5(b,c)). Comparable to Com21, we observed a negative correlation (Pearson’s ρ = −0.7878, p-value = 2.951e-06) between the biomass of the community and the growth of *C. difficile* in this community (Figure 5(d)). As expected from the higher complexity of the community and the subtler pH changes, the effect that we observed here is smaller than for Com21.

Alpha-diversity of the stool-derived community remained at a moderately high level (Shannon Diversity from 2.3 to 3) across all bioreactors over the course of the experiment with only a small drop after the 4-day change from pH 7 to the different conditions for all bioreactors (Suppl. Figure S4B). The community composition of the human stool-derived sample changed, however, especially in the pH 8 exposed bioreactors (Figure 5(e,f)). Interestingly, for all bioreactors a shift in composition, as measured by Bray-Curtis dissimilarity, was observed after the first sampling time point probably due to adjustments to the bioreactor environment (Figure 5(e)). While untreated, omeprazole treated, and pH 6 exposed communities clustered together over the course of the treatment, pH 8 exposure altered community composition differently (Figure 5(e)). At the phylum level, bioreactors at pH 8 showed increased levels of Proteobacteria (10 to 14 % increase at pH 8; 3 % decrease at pH 7 and pH 6; 2 to 5 % increase with omeprazole) and Firmicutes (4.5 to 11.5 % increase at pH 8; 0.2 to 2 % increase in the other conditions). Additionally, levels of the phyla Firmicutes_A (3 to 11.6 % decrease at pH 8; 0.8 to 3.5 % decrease in the other conditions) and Firmicutes_C (4 to 8 % decrease at pH 8; up to 4 % decrease in the other conditions) were decreased at pH 8 together with a loss of Actinobacteriota (below 0.1 %) compared to the other bioreactors (Figure 5(f)). At the family level (considering only families with at least 0.1 % relative abundance), these changes at pH 8 were mainly driven by the families *Enterobacteriaceae* (increased; Proteobacteria), *Enterococcaceae* (increased; Firmicutes), *Lachnospiraceae* (decreased; Firmicutes_A), *Peptoniphilaceae* (increased; Firmicutes_A), and *Dialisteraceae* (decreased; Firmicutes_C) (Suppl. Figure S4C). While not a main driver regarding its low relative abundance, *Ruminococcaeceae* decreased at pH 8 (relative abundance 0.03–0.09 % at pH 8 compared to 0.2–0.5 % in the other conditions). The loss of Actinobacteria at pH 8 was represented at the family level by the loss of *Coriobacteriaceae* and *Eggerthellaceae*, respectively (Suppl. Figure S4C).

Unlike for Com21 in the bioreactor, the biomass of all bioreactors with stool-derived communities did not reach the initial values after recovery, although the ability of the communities to restrict *C. difficile* growth was restored close to the baseline level (Figure 5(b,c)). This shows potential dynamics of complex communities that are missed with model communities of lower complexity.

## Discussion

CDI often occurs following antibiotic treatment, but the use of PPIs has also been associated with an increased risk of CDI. It remains unclear whether this link is due to a direct effect of PPIs on *C. difficile* or the microbiome, or if it results from altered gastrointestinal pH as a secondary effect of PPIs acting on the host. Disentangling the direct effect of the drug from the secondary pH effect is impossible in *in vivo* models or cohort studies. To address this, we used an MBS to separate the effects of the PPI omeprazole from those of altered pH on gut microbial communities. Our results showed that omeprazole does not directly affect the composition of a synthetic community of human gut commensals or its ability to limit the growth of *C. difficile*. In contrast, changes in pH were strongly correlated with altered community compositions, reduced biomass, and, ultimately, increased growth of *C. difficile* in pH-perturbed communities. These results also translated to stool-derived communities, however, with overall lower perturbation of biomass and restriction of pathogen growth. Thus, our data support the hypothesis that PPIs increase the risk for CDI by changing the pH of the gastrointestinal tract rather than by direct drug-microbe interaction.^16^ The drug-induced increase in stomach pH could directly promote the ingestion of *C. difficile* and the migration of bacteria from the upper digestive tract into the intestine. These changes may alter the microbiome composition, creating an environment favorable to *C. difficile* growth.

The gut microbiota protects against *C. difficile* through various mechanisms. These include producing inhibitory metabolites, such as secondary bile acids, short-chain fatty acids (SCFAs), and antimicrobials, as well as competition for nutrients, particularly proline and other amino acids essential for Stickland fermentation.^13,38^ In this work, we mainly focused on pH- or drug-induced changes in total biomass and community composition and their subsequent effect on *C. difficile* expansion. A previous study found that omeprazole treatment did not substantially alter the transcriptional profiles of gut bacteria, which is consistent with our finding that the drug did not have any impact on community composition or *C. difficile* expansion.^39^ However, pH disturbances may influence microbial gene expression, metabolism, or interspecies interactions in ways that promote *C. difficile* growth. Uncovering these specific molecular mechanisms, though, requires analyses beyond 16S rRNA gene amplicon sequencing.

In monocultures, a few *Bacteroidales* showed slight sensitivity to omeprazole. Still, this did not result in lower *Bacteroidales* levels in omeprazole-treated MBS communities. This contrasts with clinical studies that observed a decrease in Bacteroidetes following omeprazole treatment, which has been linked to CDI.^9,23^ Therefore, our *in vitro* results suggest that the reduction in *Bacteroidetes* seen in patients may be due to other factors than the direct inhibition of *Bacteroidales* by the drug.

Environmental factors, such as pH, strongly impact community composition due to the varying pH sensitivities of individual species. For example, *C. aerofaciens*, known for its acid tolerance,^40^ thrived in pH 5 communities. Conversely, consistent with our findings on pH sensitivity in monocultures, *Bacteroides* species exhibited acid sensitivity, and decreased in abundance in Com21 bioreactor experiments.^9,40,41^ This aligns with clinical reports of decreased *Bacteroidetes* following PPI treatment in patients,^9,23^ further supporting the notion that the association between PPIs and CDI is due to pH changes rather than a direct effect of omeprazole.

Interestingly, species like *A. rectalis*, which we identified as acid-sensitive in monoculture, thrived in pH 5 bioreactors. This aligns with previous reports of increased bacterial community abundance at pH 5.5.^36^ The seemingly contradictory discrepancy in pH sensitivity of monocultures versus bioreactor communities highlights that the presence and abundance of certain species in a community cannot be inferred solely from their individual sensitivity *in vitro*. Instead, bacterial community properties are emergent and cannot be fully explained by the sum of individual characteristics, such as pH sensitivity.

Several cohort studies reported an increase of *Enterococcaceae* and *Streptococcaceae* abundance and a decrease of *Ruminococcaceae* abundance upon PPI treatment.^10,16–18^ The increase in *Enterococcaceae* and decrease in *Ruminococcaceae* are reproducible in our experiments when increasing the pH to 8 but not with omeprazole treatment. This observation supports the hypothesis that changes in gastrointestinal pH play a more important role in PPI-induced changes in community composition than omeprazole treatment itself.

Of note, the stool samples in this study were collected from healthy individuals who had not taken proton pump inhibitors (PPIs) in the past six months. As a result, PPI-associated taxa such as *Lactobacillaceae, Micrococcaceae, Pasteurellaceae*, and *Staphylococcaceae*, which are typically low abundant in healthy individuals, were below the detection limit in our microbial communities and could not be analyzed. To investigate the effects of pH shifts and omeprazole exposure on these taxa, future studies should use samples from patients undergoing PPI treatment.

Our work focuses exclusively on vegetative cells, not on *C. difficile* spores^42^ and therefore, we examined cell proliferation rather than spore germination, assuming that the latter had already occurred. Germination of *C. difficile* spores can be triggered by small molecules, such as certain bile acids, in combination with elevated pH levels.^43^ Members of the native gut microbiota can convert primary bile acids to secondary bile acids, thereby inhibiting *C. difficile* germination. However, during dysbiosis, bile acid-converting microbiota members may be lost, leading to bile acid accumulation and subsequent germination of *C. difficile* spores.^44^ While we cannot rule out a possible effect of PPI-induced pH increases on *C. difficile* spore germination, we note that among the most prominent bile acid-converting bacteria are strains from the phyla Firmicutes, Bacteroidota, Actinomycetota, and Euryarchaeota.^42^ In the stool-derived community that we investigated in the MBS, we observed a decrease in Firmicutes A and C in the bioreactor exposed to pH 8, which could potentially promote *C. difficile* spore germination.

Our study is further limited by the inability to investigate host contributions and external factors relevant to CDI risk. Host contributions include aspects of the innate and adaptive immune responses, the host’s metabolism, as well as other host-derived factors that influence the *C. difficile* cycle, such as the enterohepatic circulation of bile acids and their interaction with the microbiome.^45^ External factors involve ectopic colonization by environmental microbes and altered food digestion due to changes in gastric conditions. Our approach also does not account for the direct effects of pH changes or omeprazole exposure on *C. difficile* virulence. Specifically, PPIs and non-physiological pH levels have been reported to increase *C. difficile* toxin expression,^46^ which is crucial for inducing colitis in patients. Thus, to thoroughly determine whether the PPI-mediated increased risk of CDI is due to potential direct interactions of omeprazole with the gut microbiome or a pH-shift dependent mechanism, further investigations are needed, including an exploration of more subtle pH changes.

Notwithstanding the aforementioned limitations, the present study offers novel insights into the potential impact of PPIs on the human gut microbiome and their correlation with an elevated risk for CDI. The investigation revealed that PPIs, such as omeprazole, do not exert a direct influence on the growth of gut microbes. Rather, these medications modify the gastrointestinal pH, thereby creating a conducive environment for *C. difficile*. This is achieved by altering the community compositions of gut microbes and weakening the low pH barrier of the stomach, which facilitates the translocation of bacteria from the upper to the lower gastrointestinal tract. Ultimately, our data suggest that any drug that increases stomach pH is likely to elevate CDI risk. Therefore, searching for new molecules that target the proton pump in parietal cells of the stomach but have fewer antibacterial effects on gut microbes is unlikely to be effective. A more promising approach would be to counteract pH changes in the large intestine and/or enhance colonization resistance through abiotic or biotic supplements.

## Methods

### Bacterial cultivation

The species and strains used in this study can be found in Supplementary Table S1. They were purchased from DSMZ and ATCC or were a gift from the Denamur Laboratory (INSERM). In the present manuscript, we use the taxonomic classification from the genome taxonomy database (GTDB) release R06–RS202.

Bacterial cultivation in monoculture was conducted as described before.^47^ In brief, all species were cultivated in modified Gifu Anaerobic Broth (mGAM) medium (HyServe GmbH & Co.KG, Germany) at 37°C except for *V. parvula*, which was grown in Todd-Hewitt Broth supplemented with 0.6 weight-% sodium lactate. The plasmid-carrying *C. difficile* strain (LM0061) was cultivated in mGAM with 15 µg/mL thiamphenicol. All media, glass, and plastic ware were pre-reduced for a minimum of 24 h under anaerobic conditions (2 vol-% H_2_, 12 vol-% CO_2_, 86 vol-% N_2_) in an anaerobic chamber (Coy Laboratory Products Inc.). Species were inoculated from frozen glycerol stocks into liquid culture medium and passaged twice (1:100) overnight before being used in subsequent experiments. To ensure no contamination of species occurred, their purity and identities were regularly checked *via* 16S rRNA gene sequencing and/or MALDI TOF mass spectrometry (MS).^48^ Stable communities from human fecal samples^49,50^ were inoculated from frozen glycerol stocks into liquid culture medium (mGAM) and incubated at 37°C overnight before being used in downstream assays. The stool-derived communities were obtained from healthy individuals, with informed consent from all donors. This study was approved by the Ethics Committee of the University Hospital Tübingen (project ID 314/2022B02). All donors confirmed that they had not taken any prescription drugs (including PPIs) in the six months prior to sample collection.

Bacterial cultivation in bioreactors was conducted with mGAM medium. Fresh mGAM medium for initial inoculation was sterilized directly in each bioreactor bottle to ensure sterility of all tubings and ports. Upon sterilization, the bioreactor bottles were sparged with N_2_ gas for at least 12 h to achieve anaerobic conditions. No growth (change in OD) after overnight incubation of the medium under a 100 vol-% nitrogen atmosphere at pH 7 and 37°C further confirmed the sterility of the system.

Each species was incubated as described above in monocultures to assemble Com18 or Com21 to inoculate the bioreactors. Afterward, the OD_600_ of every species was measured (Thermo Scientific^TM^ BioMate^TM^ 160 UV-Vis Spectrophotometer), and they were first combined at equal OD_600_ to a final OD_600_ of 0.01 so that every species contributed 0.000556 OD_600_ (Com18) or 0.000476 OD_600_ (Com21) to the culture. The bioreactors were operated in batch mode for the first 24 h to allow sufficient microbial growth. After 24 h the system was switched to continuous mode.

### pH sensitivity of individual Com21 members, C. difficile LM0061, and human stool-derived communities

To assess pH sensitivity of the community members and the plasmid-carrying *C. difficile* strain (LM0061), bacteria were grown in mGAM for two subsequent overnight cultures as described above. Human stool-derived communities were grown in mGAM for one overnight culture. Sensitivity to pH was investigated for 19 out of the 21 community members. *E. lenta* is a slow grower with poor growth in mGAM monoculture, and *V. parvula* has different media requirements in monoculture. Thus, neither was analyzed in this assay. The pH of mGAM medium was adjusted to pH 5 with hydrochloric acid, to pH 9 with sodium hydroxide, or left unchanged at pH 7.4. The pH-adjusted media were transferred to sterile Nunclon 96-well U-bottom microplates (Thermo Scientific, cat. no. 168136) inside the anaerobic chamber, and prereduced for at least 24 h. In addition to 95 µL of medium, each well was inoculated with 5 µL bacterial culture to a final OD_578_ of 0.01. Growth was measured in a plate reader over 20 h as described before^47^ and quantified by taking the maximum OD_578_ during the stationary phase and normalizing it to maximum OD_578_ at pH 7.4.

### Continuous dilution experiment of human stool-derived communities

To investigate longer exposure of human stool-derived communities to omeprazole or different pH, stool-derived communities were transferred into mGAM at pH 7.4 in deep-well plates and grown for 24 h. The next day they were diluted 1:100 into deep well plates either containing 600 µL mGAM at pH 7.4 (control), mGAM at pH 6, mGAM at pH 8, or mGAM at pH 7.4 with 80 µM omeprazole. The communities were incubated for 24 h at 37°C anaerobically before being subcultured in a 1:100 dilution again. In total, three subcultures (24 h each) were conducted at altered pH or with omeprazole before one last subculture into pH 7.4 for all communities was conducted (recovery). Before each new subcultivation, OD_578_ of all communities was measured and the relative OD to the unperturbed control wells was calculated per stool-derived community. Additionally, every community was tested for their colonization resistance against *C. difficile* (see *In vitro* invasion assay and Pathogen challenge below).

### Bioreactor handling and operating conditions

A list of all the equipment for the construction of the bioreactor system is summarized in Supplementary Table S2. The 500-mL bioreactor bottles were operated with a working volume of 250 mL. The bioreactors were continuously sparged with N_2_ at approximately 2.5 mL min^−1^ to minimize the risk of O_2_ intrusion into the system. Agitation by a magnetic stirrer was set to 200 rpm. The cultivation temperature was set to 37°C (±0.2 °C) and maintained at any time by a water thermostat circulating water through the double-walled bioreactor bottles. The starting pH was 7 (±0.05), and the hysteresis was set to 0.01. The pH probes in each bioreactor bottle were calibrated before autoclaving. The pH was measured daily with an external pH probe (pH-electrode pHenomenal® LS 221). For pH control, we used 0.5 M hydrochloric acid and sodium hydroxide base solutions (the response of the pH probe and the pumps were too slow for molarities above 0.5 M). The bioreactors were inoculated with a starting OD_600_ of 0.01. Com18 and Com21 were inoculated such that each strain equally contributed to the starting OD_600_. Upon inoculation, the bioreactors were operated in batch mode for 24 h before switching to continuous mode. For continuous mode, we used a medium feed rate of 0.1736 mL min^−1^, corresponding to an HRT of 24 h. Samples to measure the OD_600_ were taken at least every second day. Samples with an OD_600_ higher than 0.5 were diluted 1:10 prior to the measurement. The pH was continuously monitored by the internal pH probe connected to a controller, which would trigger acid or base inflow if the pH deviated ± 0.05 from 7. The temperature and the working volume were manually monitored regularly. We observed fluctuations in the bioreactor volumes in continuous mode. Those fluctuations arise from either a medium inflow faster than the outflow or *vice versa*. Small variations in the flow rate for each bioreactor occur due to differences between the cassettes on the pump head. Variations in the flow rate are likely to affect the OD by either diluting out the bacteria or providing more nutrients for faster growth, which becomes visible through changes in the OD_600_. For the omeprazole treatment of Com21 and the human stool-derived community, we dissolved 6.908 mg/ml omeprazole (TCI, CAS 73,590–58–6) in DMSO, and added 1 ml of the solution to each bioreactor, resulting in an overall concentration of 80 µM. To account for a possible effect coming from DMSO, all bioreactors which were not treated with omeprazole were treated with 1 mL DMSO. For the *C. difficile* invasion assays, we took a 2-mL sample of each bioreactor with a syringe and directly transferred the samples to anaerobic Hungate-type culture tubes (⌀16 ×125 mm, Glasgerätebau Ochs, Prod. No. 1020471) for transportation into the anaerobic chamber.

### Construction of a luminescent C. difficile reporter strain

A luminescent strain was constructed from *C. difficile* strain 630 (DSM27543; NT5083), a virulent and multidrug-resistant strain (epidemic type X), which was isolated from a hospital patient with severe pseudomembranous colitis and had spread to several other patients on the same ward in Zurich, Switzerland.^51^ To obtain a luminescent *C. difficile* reporter strain expressing sLucOPT under the control of the constitutive *fdxA* promoter (CD630_01721), the sequence upstream of (and including) the *fdxA* transcription start site^52^(CP010905.2: 234479–234578, reverse strand) was PCR-amplified from *C. difficile* 630 genomic DNA using the S7 Fusion High-Fidelity Polymerase (Mobidiag, Prod. No. MD-S7-100), HF Buffer (Mobidiag, Prod. No. MD-B704), and oligos FFO-772/FFO-773 to append NheI- and SacI-restriction sites to the resulting PCR product. The PCR fragment and the sLucOPT-encoding vector pAP24^53^ were digested with FastDigest NheI (cat. No. FD0974) and FastDigest SacI (cat. No. FD1133), purified from agarose gel, and subsequently ligated using the T4 DNA ligase (Thermo-Fisher, cat. No. 15224017), resulting in pFF-189. The plasmid was transformed to *E. coli* TOP10 for propagation, transformed to the donor strain *E. coli* CA434 (HB101 carrying the IncPb conjugative plasmid R702), and finally delivered to *C. difficile* 630 (DSM27543) by conjugation as described previously.^54^ The resulting plasmid-carrying strain, *C. difficile* [pFF-189], was designated FFS-515 (*i.e*., LM0061 in Supplementary Table S1).

### In vitro invasion assay for C. difficile

To assess the ability of *C. difficile* to grow in drug- and/or pH-treated stool-derived and bioreactor communities, we used a luminescent-based assay, which we had already established before for *Gammaproteobacteria*.^31^ Here, we used the strain *C. difficile* LM0061. *C. difficile* LM0061 was grown anaerobically in mGAM containing 15 µg/mL thiamphenicol overnight and sub-cultured (1:100) in the same medium for another overnight culture before being used in the invasion assay.

### Stool-derived bacterial communities from healthy human donors

Drug master plates in DMSO were prepared as described before,^47^ with the difference that the lowest omeprazole concentration was omitted. Instead, row E only contained DMSO and served as a control. For clindamycin, the lowest two concentrations were omitted. A 96-well deep-well plate was prepared with 95 µL mGAM per well, and 5 µL of the drug master plate was transferred into it. These plates were stored frozen for a maximum of three weeks before being used. To test the effect of different pH on bacterial communities of human fecal samples on *C. difficile* growth, 96-well deep-well plates were prepared with 475 µL of mGAM at the respective pH inside the anaerobic chamber.

Glycerol stocks from human stool samples were prepared as previously described.^47^ Anaerobic overnight cultures from glycerol stocks were directly used for the assay. The drug-mGAM deep-well plates were pre-reduced in the anaerobic chamber for 24 h before being inoculated with 400 µL human stool-derived community. Final drug concentration ranged from 2.5 µM to 160 µM for omeprazole and 10 µM to 100 µM for clindamycin with 1% DMSO and a starting human stool-derived community OD_578_ of 0.01 per well. Wells containing communities from the same donor and 1% DMSO served as controls. The pH-mGAM plates were inoculated with 25 uL of the overnight culture from stool-derived communities to a final starting human stool-derived community OD_578_ of 0.01 per well. Plates were grown for 24 h anaerobically at 37°C.

After the incubation, OD_578_ of every well was measured, and a fresh deep-well plate was prepared with 250 µL mGAM per well. Of the drug-treated- or pH-exposed human stool-derived communities, 50 µL were transferred into the fresh deep-well plate. This assay deep-well plate was used for pathogen challenge.

### Bioreactor communities

At every sampling time point, the OD_600_ of all bioreactors was measured, and a sample was transferred into a pre-reduced deep-well plate containing 250 µL mGAM (50 µL sample per well; 10 technical replicate wells per bioreactor; one plate per time point). This deep-well plate was used for pathogen challenge assay.

### Pathogen challenge (human stool-derived communities and bioreactor communities)

*C. difficile* LM0061 was diluted to an OD_578_ of 0.0025, and 200 µL were added to each well of the assay deep-well plate. The final volume was 500 µL (250 µL fresh mGAM, 50 µL drug-perturbed or pH-exposed stool-derived community/bioreactor community, 200 µL *C. difficile*) and *C. difficile* starting OD_578_ was 0.001. The assay deep-well plate was sealed with an AeraSeal breathable membrane (Sigma-Aldrich, cat. No. A9224) and incubated at 37°C anaerobically for 5 h. After incubation, all wells were thoroughly mixed, and 100 µL per well were transferred to a white 96-well plate (Thermofisher 236,105). This plate was brought out of the anaerobic chamber, and luminescence was measured with the Nano-Glo Luciferase Assay system kit from Promega (cat. No. N1110) in a Tecan Infinite 200 PRO microplate reader. The assay was done in three biological replicates.

For human stool-derived communities, the OD_578_ and luminescence were normalized to the values of the respective unperturbed controls (per donor and replicate), and the mean was calculated per drug concentration or pH, respectively.

For the bioreactor communities, the OD_600_ of every bioreactor was normalized to the median OD_600_ across all time points of the untreated control bioreactor. Luminescence data was analyzed per sampling time point. All values were normalized to the median of the untreated control bioreactor before taking the mean of all 10 technical replicates per condition and sampling time point.

### 16S rRNA gene amplicon sequencing

At every sampling point, 1 mL of the bioreactor cultures were harvested, and the pellets were frozen at −80°C for subsequent 16S rRNA gene amplicon sequencing. DNA extraction and sequencing were then conducted as described previously.^31^

In brief, DNA was isolated with the DNeasy UltraClean 96 Microbial Kit (Qiagen 10,196–4). For Com21 bioreactor samples, library preparation and sequencing were performed at the NGS Competence Center NCCT (Tübingen, Germany) with the 515F^55^ and 806 R^56^ primers (covering a ∼350-bp fragment of the 16S V4 region). Initial PCR products were purified, and indexing was performed in a second step PCR. After another bead purification, the libraries were checked for correct fragment length, quantified, and pooled equimolarly. The pool was sequenced on an Illumina MiSeq device with a v2 sequencing kit (input molarity 10 pM, 20% PhiX spike-in, 2 × 250 bp read lengths).

For stool-derived bioreactor samples, library preparation and sequencing were performed at GENEWIZ, Inc. (Suzhou, China) according to their 16S MetaVxTM method. In brief, amplicons were generated with the MetaVxTM library preparation kit (GENEWIZ, Inc., South Plainfield, NJ, USA) using the V3, V4 and V5 hypervariable region. The indexed libraries were sequenced on an Illumina MiSeq instrument (2x 250 bp read lengths).

### Computational processing of 16S rRNA gene amplicon sequences

16S rRNA analysis was conducted using the *Dieciseis* R package from our lab, which uses the standard DADA2 workflow (https://benjjneb.github.io/dada2/bigdata.html). The *Dieciseis* pipeline is optimized for the analysis of our synthetic community Com21 and is derived from the workflow described in our previous work.^31^

Briefly, quality profiles of the raw sequences were examined, trimmed, and paired-end reads filtered using the following parameters. For Com21 samples: trimLeft: 23, 24; truncLen: 225, 200; maxEE: 2, 2; truncQ: 11. For stool-derived samples: trimLeft: 16, 21; truncLen: 240, 235; maxEE: 2, 2; truncQ: 10. The filtered forward and reverse reads were dereplicated separately, and amplicon sequence variants (ASVs) were inferred using default parameters. Subsequently, the reads were merged on a per-sample basis, and the merged reads were filtered to retain only those with a length between 244 and 245 bp (for Com21) or between 393 to 403 bp and 414 to 426 bp (for stool-derived communities) before undergoing chimera removal.

For Com21 taxonomic assignment was carried out in two stages. First, the final set of ASVs was classified up to genus level using a curated DADA2-formatted database based on the genome taxonomy database (GTDB) release R06–RS202^57^ at https://scilifelab.figshare.com/articles/dataset/SBDI_Sativa_curated_16S_GTDB_database/14869077. Next, ASVs belonging to genera expected to be in Com21 were further classified at the species level using a modified version of the aforementioned database that contained only full-length 16S rRNA sequences of the 21 members of the synthetic community. The sequence of each ASV was aligned against this database using the R package DECIPHER v. 2.24.0^58^; we classified an ASV as a given species if it had sequence similarity > 98% to the closest member in the database for 20/21 species. For *V. parvula* we had to change it to > 95%. The abundance of each taxon of Com21 was obtained by aggregating reads at the species level.

For stool-derived communities only the first step of taxonomic assignment, that is ASV classification to the genus level using GTDB, was conducted.

## Supplementary Material

Supplemental Material

## Data Availability

All 16S rRNA sequencing data generated in this study is available at the European Nucleotide Archive, accession IDs PRJEB76870 and PRJEB85161.
