# Supplementary material for: Proton-pump inhibitors increase C. difficile infection risk by altering pH rather than by affecting the gut microbiome based on a bioreactor model
Source: Gut Microbes. 2025 Jun 16;17(1):2519697. doi: 10.1080/19490976.2025.2519697 (PMC12184164; doi:10.1080/19490976.2025.2519697)
Supplement: Supplemental Material [file KGMI_A_2519697_SM4937.docx]

**Proton-pump inhibitors increase *C. difficile* infection risk by altering pH rather than by affecting the gut microbiome based on a bioreactor model**

**Tables**

**Supplementary Table 1.** Strains used in this study.

| **Lab code** | **Species^1^** | **Alternative name** | **Strain** | **Source** |
| --- | --- | --- | --- | --- |
| NT5001 | *Phocaeicola vulgatus* | *Bacteroides vulgatus* | type strain | DSM No.: 1447 |
| NT5002 | *Bacteroides uniformis* |  | VPI 0061 | DSM No.: 6597 |
| NT5003 | *Bacteroides fragilis* nontoxigenic |  | EN-2,  VPI 2553 | DSM No.: 2151 |
| NT5004 | *Bacteroides thetaiotaomicron* |  | E50(VPI 5482) | DSM No.: 2079 |
| NT5006 | *Thomasclavelia ramosum* | *Clostridium ramosum* | type strain | DSM No.: 1402 |
| NT5009 | *Agathobacter rectalis* | *Eubacterium rectale* | A1-86 | DSM No.: 17629 |
| NT5011 | *Roseburia intestinalis* |  | L1-82 | DSM No.: 14610 |
| NT5017 | *Veillonella parvula* |  | type strain | DSM No.: 2008 |
| NT5024 | *Eggerthella lenta* |  | type strain | DSM No.: 2243 |
| NT5025 | *Fusobacterium nucleatum* ssp. nucleatum |  | type strain | DSM No.: 15643 |
| NT5026 | *Enterocloster bolteae* | *Clostridium bolteae* | type strain | DSM No.: 15670 |
| NT5032 | *Sarcina perfringens* | *Clostridium perfringens* | C36 | DSM No.: 11782 |
| NT5037 | *Lacrimispora saccharolytica* | *Clostridium saccharolyticum* | type strain | DSM No.: 2544 |
| NT5038 | *Streptococcus salivarius* |  | type strain | DSM No.: 20560 |
| NT5046 | *Ruminococcus*_B *gnavus* | *Ruminococcus gnavus* | type strain | ATCC No.: 29149 |
| NT5048 | *Bariatricus comes* | *Coprococcus comes* | type strain | ATCC No.: 27758 |
| NT5071 | *Parabacteroides merdae* |  | VPI T4-1, CIP 104202T | DSM No.: 19495 |
| NT5072 | *Streptococcus parasanguinis* |  | type strain | DSM No.: 6778 |
| NT5073 | *Collinsella aerofaciens* |  | type strain | DSM No.: 3979 |
| NT5076 | *Dorea formicigenerans* |  | VPI C8-13 | DSM No.: 3992 |
| NT5078 | *Escherichia coli* |  | ED1α | Denamur Lab (INSERM) |
| LM0061^2^ | *Clostridioides difficile* |  | FFS-515, Cm^R^; carrying pFF189 | Faber lab (Uni Würzburg) |

^1^ taxonomic classification based on the genome taxonomy database (GTDB) release R06-RS202

^2^ based on strain 630; Cm^R^, chloramphenicol acetyl-transferase for chloramphenicol/thiamphenicol selection

**Supplementary Table 2.** Materials for the MBS.

| **Item** | **Reference number** | **Supplier** | **Quantity** |
| --- | --- | --- | --- |
| Heating thermostat (CC-104A) | 461-1056 | HUBER *via* VWR | 1 |
| Masterflex L/S® Multichannel Cartridge Pump Head with Reduced Pulsation for Microbore 2-Stop Tubing, 12-Channel, 8-Roller | HV-07519-25 | Cole-Parmer | 1 or 2 |
| Masterflex L/S® Small Cartridges for Multichannel Cartridge Pump Head with Reduced Pulsation for Microbore 2-stop Tubing | SI-07519-85 | Cole-Parmer | 12 |
| DURAN® double walled, wide mouth bottle GLS 80®, 500mL | 215-4156 | VWR | 6 |
| BOLA GLS 80 Vessel Closure \| PTFE \| 5 x GL 14 \| 1 x GL 25 | XZ019-182117 | Zinsstag | 6 |
| Multi-position magnetic stirrers, MIX series | 442-0752 | VWR | 1 |
| Masterflex L/S® Variable-Speed Digital Drive with Remote I/O, 1 to 100 rpm; 90 to 260 VAC | HV-07528-30 | Cole-Parmer | 1 or 2 |
| Masterflex C/L® Analog Variable-Speed Pump with Single-Channel Pump Head for Microbore Tubing Pump, 13 to 80 rpm; 90 to 260 VAC | 77122-32 | Cole-Parmer | 12 |
| MV5010 pH / Redox / ISE-Transducer with display in wall-mounting case | 90278080 | Xylem/Si-analytics | 6 |
| Item stand for MBS | Offer Nr.: AN00178860-1 | ITEM | 1 |
| Screws for MBS system | 6834886 | Hornbach | 1 pck |
| Power strip | 3882931 | Hornbach | 2 |
| Tygon tubing A-60-G, I/P 73, 50 ft. | MFLX06404-73 | VWR | 2 |
| Flexible Cable H05VV-F 4 x 1 mm², black, sold by meter | 1499067 | Conrad | 10m |
| Ferrule 1 mm² Partially Insulated | 617836 - 62 | Conrad | 1 pck |
| Octagon 8 port - manifold | 343938 | Huber | 1 |
| Masterflex® Ismatec® Pump Tubing, 2-Stop, Viton®, 2.06 mm ID, 15" L; 12/PK | MFLX96428-42 | VWR | 1 |
| Laboratory Screw Joints, GL14 cap, 3-parts, including PTFE/ETFE fittings (6mm) | D590-06 | Bola | 30 |
| Laboratory Screw Joints, GL25 cap, 3-parts, including PTFE/ETFE fittings (12mm) | D590-34 | Bola | 6 |
| Hose connectors ROTILABO® Y-shape with conical ends, hose inner Ø 9-11mm | TT53.1 | Carl Roth | 1 pck |
| Hose connectors ROTILABO® T-shape, Hose inner Ø 10-11 mm | E767.1 | Carl Roth | 1 pck |
| Hose connectors ROTILABO® angle shape, Hose inner Ø 10-11 mm | E788.1 | Carl Roth | 1 pck |
| Rapid couplings, male connections, hose nipples with hose nozzles, Ø9.5mm | 8771-1060 | Bürkle | 8 |
| ATEX II 1/2G pH-single-rod measuring cell | SL 81-225 pHT VP | Xylem/Si-analytics | 6 |
| Variopin cable | 85442000 | Xylem/Si-analytics | 6 |
| Disposable needles Sterican® long bevel facet, 30 mm, 0.60 mm, blue | X129.1 | Carl Roth | 6 per experiment |
| Button Once Canulla, 45 mm length | KK45R21S | CLS Medizintechnik und Vertrieb | 6 per experiment |
| Gas trap | 114100 | Ochs | 6 |
| Pump Tubing, PharMed® BPT, 1,14 mm ID; 100 ft | MFLX95809-30 | VWR | 1 pck |
| Masterflex L/S Norprene Food-Grade Tubing, L/S 16, 50ft. | MFLX06402-16 | VWR | 2 pck |
| Magnetic bars ROTILABO® Economy, Ø: 8 mm, 25 mm | XA18.1 | Carl Roth | 6 |
| Set of customized stainless-steel tubing, Ø  6mm, tube (240 mm) with sparger, sample  tubing (200 mm), off-gas tubing (100 mm) | Offer Nr.: BZV-2022111600511 | bbi-biotech | 6 of each |
| 5 L Tedlar® Gas sampling bags, 2-in-1 PP valve Thermogreen® LB-2 Septa | 24655 | VWR | 1 |
| J.T.Baker®, Syringe Filters, pore size 0,22 µm | SF02-60 | VWR | 100 |
| Luer/Lock fittings in different sizes | CT59.1-64.1 | Carl Roth | 150 |
| 5-L duran bottle | 215-0057 | VWR | 2 |
| 1-L duran bottle | 215-1595 | VWR | 2 |

**Supplementary Figure 1.**  **Luminescence of *C. difficile* reflects bacterial numbers and remains at similarly low levels in untreated Com21 and stool-derived communities compared to monoculture. A)** Growth curves for *C. difficile* LM0061 based on plating on mGAM agar (top) or luminescence (bottom). The lines indicate the mean of three biological replicates. Red vertical lines mark the endpoint of the *C. difficile* invasion assay, which falls within the linear range of the curves, allowing luminescence to be used as a proxy for *C. difficile* levels. **B)** Relative growth of *C. difficile* during co-culture with untreated Com21 from bioreactor 5 or human stool-derived communities in vitro or human stool-derived samples in the bioreactor compared to pure culture. Pathogen levels were quantified via luminescence after 5 h. For Com21 and stool-derived samples in the bioreactor, values are shown from each sampling point of the multiple-bioreactor system (six or five in total) from bioreactor 5 (pH 7, untreated) or bioreactor 1 (pH 7, untreated), respectively. For human stool-derived communities, the mean of three biological replicates is shown per fecal sample. Red points and bars represent mean (M) ± standard error of the mean (SEM). No significant (ns) difference in relative *C. difficile* growth between Com21 from bioreactor 5 (M = 0.0158, SEM = 0.0019), human stool-derived communities (M = 0.0179, SEM = 0.001), and the human stool-derived community in the bioreactor (M = 0.0138, SEM = 0.0004). One-sided ANOVA: F(2,16) = 2.45; p = 0.118.


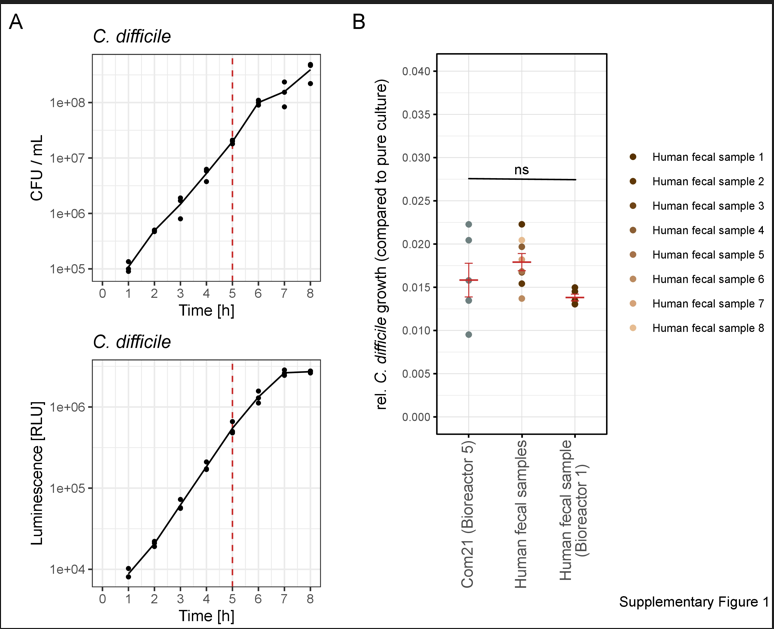


**Supplementary Figure 2. Continuous dilution of human stool-derived communities with omeprazole treatment or altered pH does not alter biomass nor promote the growth of *C. difficile*.** A) Bacterial communities derived from eight human fecal samples were grown in deep well plates in mGAM at pH 7.4 for 24 h. After that they were diluted 1:100 every 24 h into fresh medium at pH 7.4 (control), pH 6, pH 8, or pH 7.4 with 80 µM omeprazole for three days. After 72 h of treatment or altered pH exposure all communities were diluted into mGAM at pH 7 again (Recovery). The top plot shows the community biomass as measured by optical density (OD) relative to the unperturbed control communities at pH 7.4. The lower plot shows the mean log2 fold change in *C. difficile* growth relative to the unperturbed control communities at pH 7.4. Red horizontal lines depict the mean per perturbation time point (N = 3).


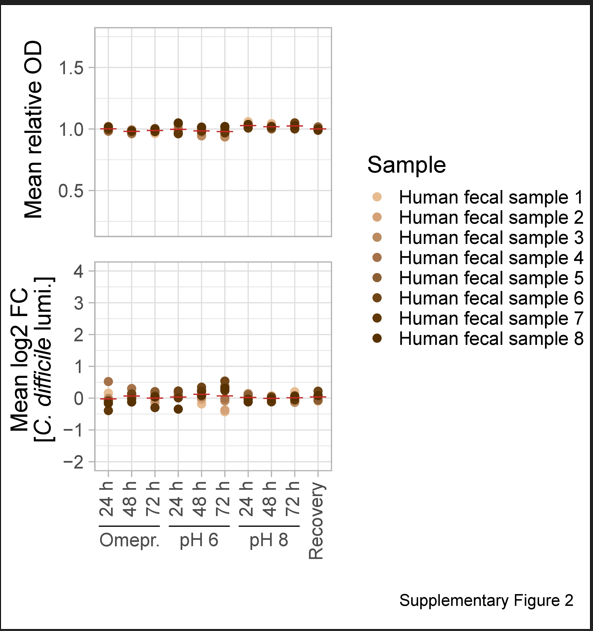


**Supplementary Figure 3. Continuous growth of Com18 in the MBS. A)** OD of the bioreactors throughout the entire operation period. The community was grown in batch mode for one day before switching to continuous mode (indicated by the gray dashed line). **B)** Relative abundance of each strain in the Com18 at the indicated time points. Triplicates are presented in one panel. The first two sampling time points were during batch mode, while the subsequent sampling time points were during continuous mode.


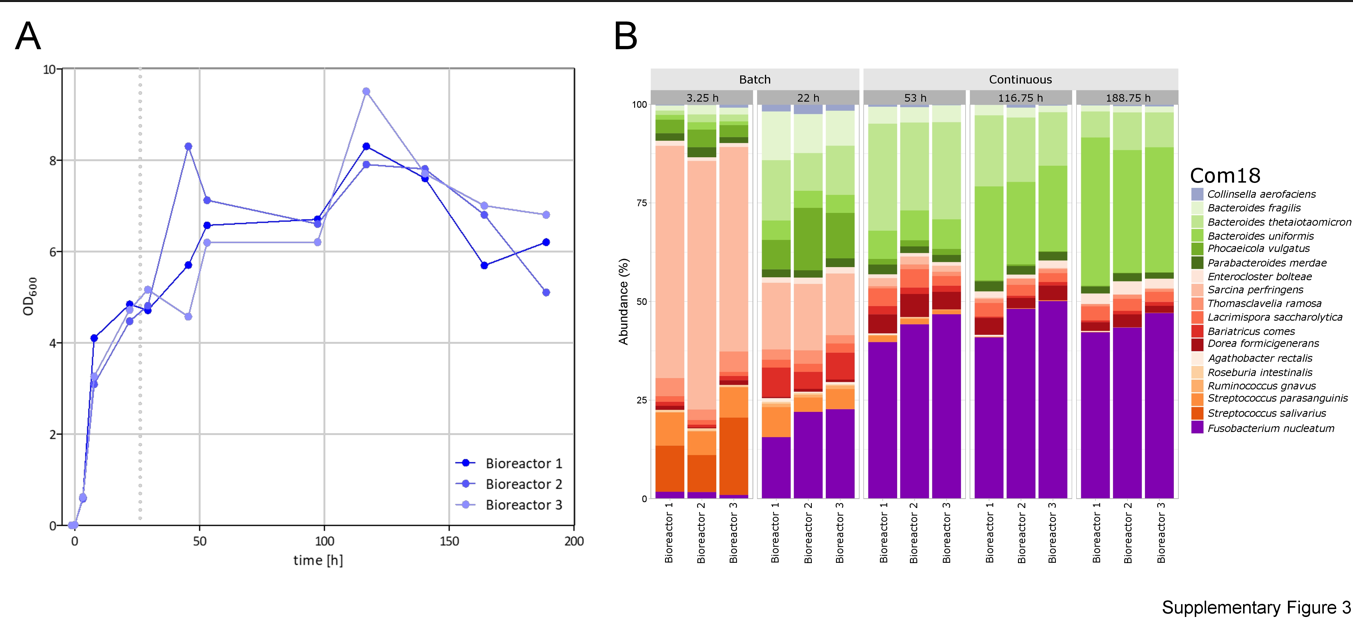


**Supplementary Figure 4. Composition and alpha-diversity of the eight human fecal samples**. A) Relative abundances of the human fecal samples grown in mGAM at the phylum level. B) Shannon diversity of human fecal sample 1 over time in the bioreactors with different conditions. C) Relative abundance (at family level) of human stool-derived sample 1 in the bioreactors at the indicated sampling time points. Bacterial families were filtered to have a relative abundance of at least 0.1 %. Panels are grouped by bioreactor: Bioreactor 1 was left untreated, bioreactors 2 and 5 were treated with omeprazole, bioreactor 4 was changed to pH 6, and bioreactors 3 and 6 were changed to pH 8.

**
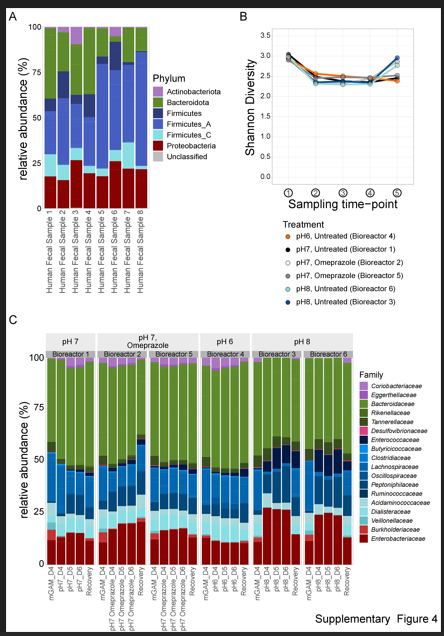
**
